# Splenic, Nonhairy, B‐Cell Lymphoma Presenting as Fever of Unknown Origin

**DOI:** 10.1155/carm/4919763

**Published:** 2026-05-08

**Authors:** Abdul Rehman, John E. McKnight, Hafiz Javed, Arfa Ahmad, Patrick O’Reilly

**Affiliations:** ^1^ Department of Medicine, TidalHealth Peninsula Regional, Salisbury, Maryland, 21801, USA; ^2^ Department of Pathology, TidalHealth Peninsula Regional, Salisbury, Maryland, 21801, USA

**Keywords:** fever of unknown origin, hairy cell leukemia-variant, splenic B-cell lymphoma with prominent nucleolus

## Abstract

Fever of unknown origin (FUO) is a common diagnostic dilemma faced by internists on a fairly regular basis. A wide variety of infectious, autoimmune, and neoplastic conditions can present with FUO. Splenic B‐cell lymphoma/leukemia with prominent nucleolus (SBLPN), formerly known as hairy cell leukemia‐variant, is a hematologic malignancy that often presents with massive splenomegaly and pancytopenia. Initial presentation of SBLPN as FUO is exceedingly rare. Here, we present the case of an elderly gentleman in his 80s who presented with fever, night sweats, weight loss, and recurrent falls. He was subsequently diagnosed with SBLPN after 6 weeks of extensive diagnostic evaluation. The practicing internist needs to be aware of this initial presentation of SBLPN as FUO and have a low suspicion to proceed with a bone marrow aspiration and biopsy to clench the diagnosis in the right clinical context.

## 1. Introduction

Fever of unknown origin (FUO) is a common diagnostic dilemma faced by internists on a regular basis [1]. A wide variety of infectious, autoimmune, and neoplastic conditions can present with FUO [2, 3]. Hematologic malignancies in general, and splenic lymphomas in particular, can rarely present with FUO. Hairy cell leukemia (HCL) and related splenic B‐cell lymphoid neoplasms comprise a diagnostically challenging group of disorders with overlapping clinical, morphologic, immunophenotypic, and molecular features [4, 5]. Classic HCL typically presents with splenomegaly, cytopenias, and circulating lymphoid cells with cytoplasmic projections (so‐called “hairy cells”) and is characteristically associated with the expression of CD11c, CD25, CD103, CD123, annexin A1, and the BRAF V600E mutation. In contrast, splenic B‐cell lymphoma/leukemia with prominent nucleoli (SBLPN), previously termed HCL‐variant, is a distinct entity with different biologic behavior, immunophenotypic features, and therapeutic implications. Compared with classic HCL, SBLPN more often shows prominent nucleoli, lacks CD25 and annexin A1 expression, and follows a more aggressive clinical course with less favorable response to conventional purine analog therapy. The differential diagnosis of SBLPN also includes splenic diffuse red pulp small B‐cell lymphoma (SDRPL) and splenic marginal zone lymphoma (SMZL). SDRPL is characterized by diffuse involvement of the splenic red pulp, whereas SMZL is a postgerminal center B‐cell neoplasm involving the splenic white pulp and marginal zone. Because these entities can be difficult to distinguish on clinical grounds alone, accurate diagnosis requires the integration of morphology, flow cytometry, immunohistochemistry, and molecular findings. The incidence of SBLPN has been estimated to be less than 1 in 100,000 person‐years in the United States [5]. Initial presentation of SBLPN as a FUO is exceedingly rare. Here, we present the case of an elderly gentleman in his 80s, who presented with fever, night sweats, weight loss, and recurrent falls and was subsequently diagnosed with SBLPN after 6 weeks of extensive diagnostic evaluation.

## 2. Case Presentation

An elderly gentleman in his 80s initially presented to the emergency department of our institution with complaints of fever, night sweats, and loss of appetite for 3 weeks. He denied any symptoms of cough, sputum production, shortness of breath, urinary complaints, or alteration in bowel habits. He did endorse having some unintentional weight loss but could not quantify the exact amount. He denied any complaints of easy bruising, gum bleeding, fatiguability, or any lumps in his neck, axilla, or groin. His past medical history was remarkable for essential hypertension, hyperlipidemia, chronic kidney disease stage 3A, paroxysmal atrial fibrillation, history of ischemic stroke (without residual neurologic deficits), and gastroesophageal reflux disease with reflux esophagitis and Barrett’s esophagus. His past surgical history was remarkable for transurethral resection of the prostate for benign prostatic hyperplasia. His family history was notable for a history of melanoma in his father and a history of brain aneurysm in his mother. He never smoked cigarettes and denied any illicit drug use. He drank a glass of wine with dinner daily. He lived in a woody, suburban area of Northern America and reported traveling to the Caribbean Islands about 4 months prior to current presentation. He did take frequent hikes in the forest but denied any history of tick bites. He did not have any known history of drug allergies. His regular medications included atorvastatin, ferrous sulfate, lisinopril, omeprazole, and warfarin.

On initial evaluation, his vital signs were only notable for bradycardia (pulse rate 59 beats per minute, irregularly irregular) and borderline low blood pressure (109/48 mm·Hg). His physical examination revealed an elderly gentleman of average built lying in bed in no acute distress. He had pale conjunctivae but anicteric sclera. His mucous membranes were moist and pharynx was devoid of exudate or erythema. Cervical, axillary, and inguinal lymph nodes were not enlarged. His lungs were clear to auscultation, and his cardiac auscultation was notable for a faint (Grade 2/6) ejection systolic murmur at the aortic area as well as irregularly irregular rhythm. Apical impulse was normal in character and nondisplaced. His abdominal examination was notable for splenomegaly (splenic tip felt 6 cm below the left costal margin) although the spleen was nontender and without any bruit. He had bilateral +2 pitting pedal edema. Skin examination revealed multiple scattered solar lentigines as well as seborrheic keratoses and bruises over both arms.

**FIGURE 1:**
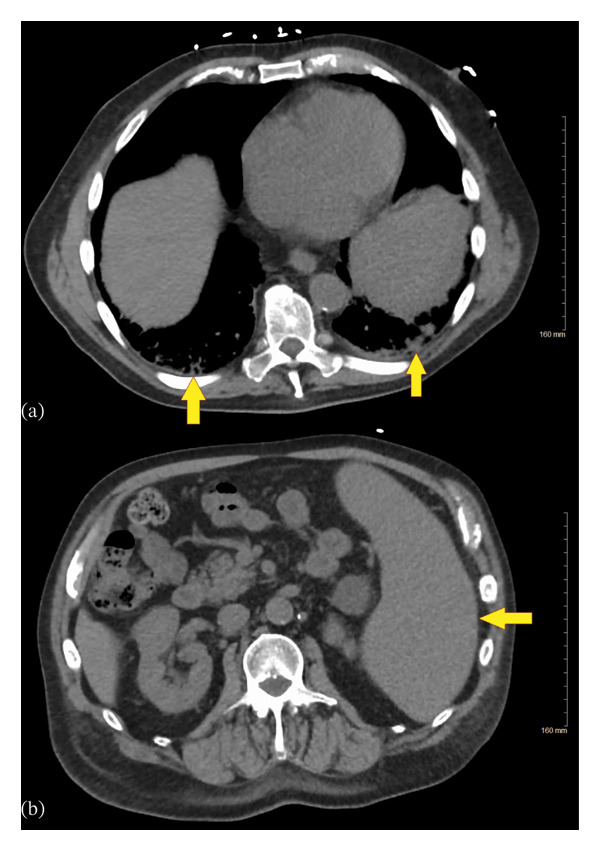
Computed tomography of the chest and abdomen in our patient on initial presentation. (a) Axial section at lower thoracic level demonstrating bibasilar infiltrates and atelectasis (arrows). (b) Axial section at upper abdominal level demonstrating splenomegaly (arrow).

The results of all laboratory investigations are provided in Table 1. His complete blood count on presentation was remarkable for leukocytosis (total leukocyte count: 22.1 × 10^9^/L) with absolute lymphocytosis (53% lymphocytes) and monocytosis (16% monocytes), normochromic normocytic anemia (hemoglobin: 8.6 g/dL), and thrombocytopenia (platelet count: 51 × 10^9^/L) with a normal mean platelet volume (MPV: 8.1 fL). His urea, creatinine, electrolytes, and creatine kinase were within normal limits. His urine dipstick and microscopy were also unremarkable. His chest radiograph did not reveal any pulmonary infiltrates. Initially, he was kept on piperacillin–tazobactam and vancomycin for possible sepsis. CT scans of the head, chest, abdomen, and pelvis were performed to reveal any occult focus of infection. CT chest was only notable for bilateral basal atelectasis (Figure 1(a)), and CT abdomen was remarkable for splenomegaly (Figure 1(b)). His blood cultures revealed no growth. Given his travel history, malaria smears were also performed, which were negative. A tick‐borne illness panel (*Anaplasma*, *Ehrlichia*, *Borrelia*, and *Babesia*) was sent, and the patient was empirically switched to amoxicillin–clavulanate and doxycycline for possible pneumonia or tick‐borne illness. The patient was discharged home with a plan to follow‐up as outpatient.

**TABLE 1:** Results of laboratory investigations.

Investigation	Result	Reference range
Hematology		
Hemoglobin	8.6 g/dL	12.0–16.5 g/dL
Hematocrit	26.5%	36.0%–50.0%
Mean corpuscular volume	88 fL	80–96 fL
Red blood cell count	3.01 × 10^12^/L	4.1–5.3 × 10^12^/L
Red cell distribution width	26.2%	12.3%–17.0%
Total leukocyte count	22.1 × 10^9^/L	3.6–11.2 × 10^9^/L
Neutrophils	31%	41%–73%
Lymphocytes	53%	19%–45%
Monocytes	16%	5%–12%
Platelet count	51 × 10^9^/L	150–400 × 10^9^/L
Mean platelet volume	8.1 fL	7.4–10.4 fL
*Coagulation*		
Prothrombin time	24.8 s	9.4–12.5 s
INR	2.1^∗^	0.9–1.2
D‐dimer	8.51 mcg/mL FEU	< 0.5 mcg/mL FEU
*Chemistry*		
Sodium	136 mmol/L	136–144 mmol/L
Potassium	3.8 mmol/L	3.6–5.1 mmol/L
Chloride	105 mmol/L	101–111 mmol/L
Bicarbonate	22 mmol/L	22–32 mmol/L
Blood urea nitrogen	28 mg/dL	8–23 mg/dL
Creatinine	1.1 mg/dL	0.64–1.27 mg/dL
Blood glucose	103 mg/dL	80–140 mg/dL
Creatinine kinase	62 U/L	49–397 U/L
*Liver function tests*		
Alanine aminotransferase	34 IU/L	17–3 IU/L
Aspartate aminotransferase	51 IU/L	15–41 IU/L
Total bilirubin	1.4 mg/dL	0.3–1.2 mg/dL
Alkaline phosphatase	184 U/L	56–155 U/L
Total protein	5.6 g/dL	6.1–7.9 g/dL
Albumin	2.0 g/dL	3.2–5.0 g/dL
*Other (miscellaneous)*		
Serum viscosity	1.0 cpoise	≤ 1.5 cpoise
Vitamin B_12_	386 pg/mL	193–986 pg/mL
Ferritin	324 ng/mL	24–336 ng/mL
Haptoglobin	114 mg/dL	30–200 mg/dL
*Autoimmune workup*		
Antinuclear antibody	Negative	—
Anti‐dsDNA antibody	Negative	—
Anti‐MPO antibody	Negative	—
Anti‐PR3 antibody	Negative	—
*Infectious workup*		
Blood culture	No growth	—
HIV Ag/Ab combo	Nonreactive	—
Hepatitis A IgM	Nonreactive	—
Hepatitis B surface antigen	Nonreactive	—
Hepatitis B surface antibody	Nonreactive	—
Hepatitis B core antibody	Nonreactive	—
Hepatitis B e antigen	Nonreactive	—
Hepatitis C antibodies	Nonreactive	—
Malaria smear	Negative	—
RPR	Negative	—
*Histoplasma* CF/ID	Negative	—
CMV PCR	Negative	—
EBV VCA IgM	Negative	—
EBV VCA IgG	Positive	—
*Toxoplasma* IgM	Negative	—
*Brucella* IgM ELISA	Negative	—
*Brucella* IgG ELISA	Negative	—
*Ehrlichia chaffeenis* IgM	< 1:20	< 1:20
*Ehrlichia chaffeenis* IgG	< 1:64	< 1:64
Lyme antibodies	Negative	—
RMSF IgM	Negative	—
RMSF IgG	Negative	—
*Anaplasma* IgM	< 1:20	< 1:20
*Anaplasma* IgG	< 1:64	< 1:64
*Babesia microti* IgM	< 1:20	< 1:20
*Babesia microti* IgG	< 1:64	< 1:64
*Babesia duncani* IgG	< 1:256	< 1:256
*Babesia divergens* IgG	Negative	—
*Coxiella burnetti* antibody	Negative	—
*Francisella tularensis* IgM	Negative	—
*Francisella tularensis* IgG	Negative	—
*Bartonella* spp. antibodies	Negative	—
*Cerebrospinal fluid studies*		
Appearance	Clear	—
Leukocytes	2	< 5 cells/mm^3^
Erythrocytes	6	None
Glucose	59 mg/dL	40–70 mg/dL
Protein	31 mg/dL	15–45 mg/dL
Gram stain	Negative	—
Bacterial culture	No growth	—
Fungal culture	No growth	—
AFB stain and culture	Negative	—
Cytology	No malignant cells	—
*Escherichia coli* K1, PCR	Not detected	
*Hemophilus influenzae* K1, PCR	Not detected	
*Listeria monocytogenes*, PCR	Not detected	
*Neisseria meningitidis*, PCR	Not detected	
*Streptococcus agalactiae*, PCR	Not detected	
*Streptococcus pneumoniae*, PCR	Not detected	
CMV PCR	Not detected	
Enterovirus PCR	Not detected	
Human herpesvirus 6 PCR	Not detected	
Herpes simplex virus‐1 PCR	Not detected	
Herpes simplex virus‐2 PCR	Not detected	
Human parechovirus, PCR	Not detected	
Varicella zoster virus, PCR	Not detected	
*Cryptococcus neoformans*, PCR	Not detected	
La Crosse encephalitis, IgG	< 1:1	
La Crosse encephalitis, IgM	< 1:1	
East equine encephalitis, IgG	< 1:1	
East equine encephalitis, IgM	< 1:1	
St. Louis encephalitis, IgM	< 1:1	
St. Louis encephalitis, IgG	< 1:1	
West equine encephalitis, IgM	< 1:1	
West equine encephalitis, IgG	< 1:1	

*Note:* CMV = cytomegalovirus; ELISA = enzyme linked immunosorbent assay; MPO = myeloperoxidase; PR3 = proteinase 3.

Abbreviations: AFB = acid‐fast bacilli, CF/ID = complement fixation/immunodiffusion, EBV = Epstein–Barr virus, HIV = human immunodeficiency virus, INR = international normalized ratio, RMSF = Rocky Mountain spotted fever, RPR = rapid plasma reagin.

^∗^Patient was on warfarin therapy with target INR 2‐3.

Based on the patient’s initial presentation with recurrent fevers, night sweats, weight loss, and splenomegaly, the differential diagnosis was very broad—including infectious etiologies, neoplastic diseases, and rheumatologic conditions. At the time of first hospitalization, the working diagnosis was thought to be a tick‐borne illness with a probable component of superimposed bacterial pneumonia. The diagnosis of tick‐borne illness was presumptive and based on the patient’s history of taking frequent hikes and living in a woody, suburban area. Moreover, the diagnosis of superimposed bacterial pneumonia was considered in view of leukocytosis and subtle basal pulmonary infiltrates.

Despite taking the antibiotics, the patient continued to spike fevers about every 48–72 h. He also endorsed having generalized weakness and suffered a fall at home. The results of his tick‐borne illness panel came back negative. At this time, the differential diagnosis included other infectious diseases (such as tuberculosis, brucellosis, Pel–Ebstein fever, toxoplasmosis, and histoplasmosis), rheumatic diseases (such as Adult‐onset Still’s disease and ANCA vasculitis), and neoplastic diseases (occult malignancies). Therefore, he was rehospitalized for further evaluation of FUO.

On the second hospitalization, the infectious diseases team was consulted and extensive workup for infectious illnesses was sent (see Table 1). An autoimmune panel (antinuclear antibody, antineutrophil cytoplasmic antibody, and rheumatoid factor) was also sent, which resulted negative. Infectious workup came back negative for Ebstein–Barr virus, cytomegalovirus, human immunodeficiency virus, hepatotropic viruses, *Brucella*, *Histoplasma*, *Coxiella*, *Francisella*, *Bartonella*, *Toxoplasma*, and syphilis. A lumbar puncture was also performed, which revealed normal cell count, glucose, and protein levels.

**FIGURE 2:**
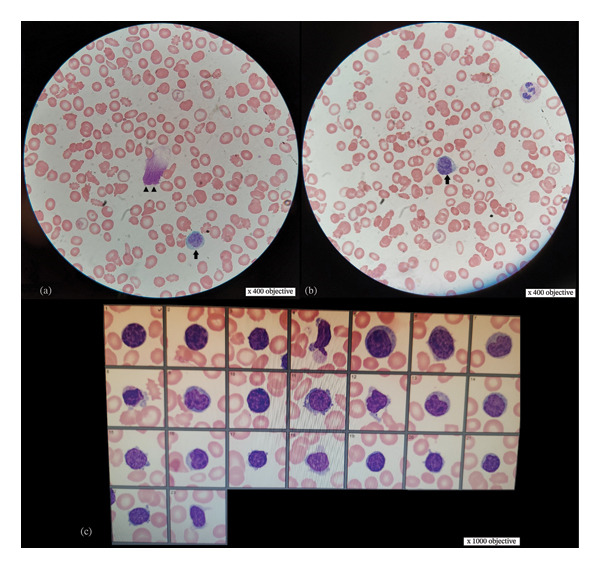
Peripheral smear for our patient. (a‐b) Wright–Giemsa stains (× 400 magnification) demonstrate anisocytosis, dacrocytes (teardrop cells), acanthocytes, smudge cell (arrowheads), and atypical lymphocytes (arrows) with coarse chromatin and basophilic cytoplasm. (c) Automated images of lymphocytes acquired by Beckman Coulter Hematology Analyzer CellaVision (Beckman Coulter, Inc.; Brea, California, United States) at × 1000 magnification. Multiple atypical lymphocytes with coarse chromatin, basophilic cytoplasm, and occasional cytoplasmic projections are seen.

**FIGURE 3:**
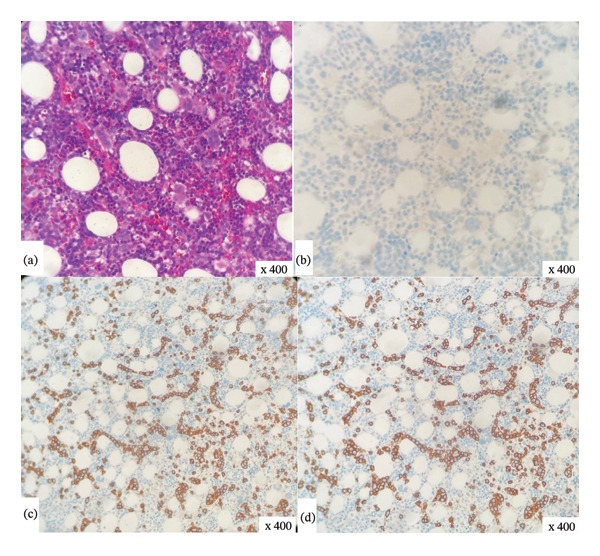
Slides of bone marrow aspiration and biopsy for our patient. (a) Hematoxylin and eosin stain (× 400 magnification) demonstrating a hypercellular marrow. (b) Absence of staining with antibody directed against the mutant BRAF V600E protein (× 400 magnification). (c‐d) CD20 immunostains demonstrating linear array of B lymphocytes in a sinusoidal pattern (× 400 magnification).

At this point, the hematology team was consulted and a plan was made for a bone marrow aspiration and biopsy. Peripheral blood flow cytometry for lymphocytes was also performed, which revealed a monoclonal population of B‐cells with kappa (*κ*) light‐chain restriction. B‐cells were positive for CD19, CD20, CD11c/CD22, and CD103—similar to HCL—but negative for CD200—suggestive of HCL‐variant. Peripheral smear review by a hematopathologist was notable for numerous atypical lymphocytes and smudge cells (Figure 2). Bone marrow aspiration and biopsy (Figure 3) revealed hypercellular marrow, normochromic normocytic anemia with marrow erythroid hyperplasia, and thrombocytopenia with marrow megakaryocytic hyperplasia—consistent with peripheral sequestration or destruction. Moreover, the monoclonal proliferation of B‐cells was also detected (immunophenotypically identical to peripheral blood) with intrasinusoidal infiltration; these B‐cells were negative for annexin A1, SOX11, and LEF1. Moreover, B‐cell subpopulation was also negative for BRAF V600E and MYD88 L265P mutations. Results of cytogenetic analyses revealed near‐tetraploidy in 65% of nuclei along with *TP53* deletion and 7q13 microdeletion. Based on the clinical, morphological, cytogenetic, and immunophenotypic characteristics of the monoclonal B‐cells along with the patient’s overall clinical picture, a diagnosis of splenic, nonhairy, B‐cell lymphoma was made. Within the 5^th^ edition of the World Health Organization classification of hematolymphoid tumors (WHO‐HAEM5), splenic histology is necessary for unequivocally differentiating SBLPN from SMZL and splenic diffuse red pulp lymphoma. As such, this patient’s probable disease (SBLPN) could not be differentiated from SMZL or splenic diffuse red pulp lymphoma on clinical grounds alone. Moreover, in this patient, the risks of splenic biopsy or splenectomy were thought to outweigh the benefits—especially since the patient’s treatment would be the same regardless of the results of splenic biopsy.

After a diagnosis of splenic, nonhairy, B‐cell lymphoma was made, the patient was planned for the initiation of weekly single‐agent rituximab 375 mg/m^2^ infusions in a split‐dose (2‐day) schema for 4–6 weeks. Unfortunately, the patient developed Grade 4 Type I hypersensitivity reaction during the first infusion of rituximab. This reaction was characterized by hoarseness, diffuse polyphonic wheezing, alteration in mental status, and hives. The patient was promptly treated with intramuscular epinephrine, intravenous methylprednisolone, and intravenous diphenhydramine with improvement in his symptoms. Patient was then given cladribine 0.1 mg/kg IV once daily for 5 days. He tolerated the cycle of cladribine without any adverse effects and had normalization of his white blood cell count by Day 10 of Cycle 1. His fever had resolved after the initiation of chemotherapy, and he was discharged to inpatient rehabilitation facility.

After completing inpatient rehabilitation, patient continued to follow‐up in the oncology clinic. After securing insurance approval, the patient was started on chemotherapy with obinutuzumab every 4 weeks for 6 cycles. Patient tolerated 6 cycles of obinutuzumab well, and a repeat CT chest, abdomen, and pelvis with contrast showed normal‐sized spleen and no residual lymphadenopathy. At this point, maintenance therapy with obinutuzumab every 12 weeks was started. At the time of last follow‐up (18 months after initial diagnosis), the patient was tolerating obinutuzumab well and feeling in excellent state of health.

## 3. Discussion

FUO is a common diagnostic challenge faced by internists and requires a tailored diagnostic approach for each patient based on their presenting clinical features and medical characteristics [1–3]. FUO has been traditionally defined as documented spikes of fever (temperature > 38.3°C) on several occasions over a 3‐week period without a clear etiology identifiable after an intensive 1‐week investigation in the hospital [2]. Previously, a classification of FUO into classic, nosocomial, neutropenic, and HIV‐related FUO was proposed with the hopes of identifying distinct subgroups of patients with similar etiologies of FUO [6]. However, even with this classification, there is no single unifying algorithm that can fit the evaluation of each patient with FUO [1–3, 7]. As such, each patient with FUO requires careful history taking, physical examination, and a tailored workup to delineate the etiology underlying FUO. In our case, extensive infectious workup was undertaken in view of the presence of long‐standing fevers, splenomegaly, thrombocytopenia, anemia, and hypoalbuminemia without any lymphadenopathy or hepatomegaly. In cases where a lymph node is accessible for percutaneous biopsy, this can greatly assist in narrowing the differential diagnosis [8]. However, splenic biopsy is typically avoided due to its high vascularity and risk for hemorrhage [9]. In our case, bone marrow aspiration and biopsy along with peripheral blood flow cytometry were instrumental in establishing the diagnosis of SBLPN. In a patient with gross abnormalities on the complete blood count with differential, a review of the peripheral smear by a qualified hematopathologist is dispensable as it can identify the presence of atypical lymphocytes, smudge cells, or blast cells. Moreover, in any patient with absolute lymphocytosis, flow cytometry with immunophenotyping is essential to exclude a monoclonal proliferation of lymphocytes.

SBLPN was a placeholder category introduced in WHO‐HAEM5 to replace CD5‐negative B‐cell prolymphocytic leukemia and HCL‐variant [4]. HCL‐variant was first described in 1980 in two patients with circulating villous cells in the blood with prominent nucleoli and massive splenomegaly in the absence of any myelofibrosis or monocytopenia [5]. This disease entity was associated with a distinct immunophenotype of B‐cells as well as a worse prognosis when compared with classic HCL. The main differentiating features of HCL‐variant were considered to be the absence of BRAF V600E mutation, negative annexin A1 staining, and negativity for CD25 in conjunction with copositivity for CD20 and CD11c [10]. Subsequently, HCL‐variant was reclassified as SBLPN in WHO‐HAEM5 classification based on the presence of neoplastic B‐cells with a single prominent nucleolus, abundant basophilic cytoplasm, and variable cytoplasmic projections [4]. In our case, patient’s B‐cells were negative for BRAF V600E mutation as well as negative for annexin A1, LEF1, and SOX11. Interestingly, the neoplastic cells were intermediate‐sized with round to irregular nuclei, reticulated chromatin, pale staining cytoplasm, and inconspicuous nucleoli. Splenomegaly was present along with the absence of lymphadenopathy or hepatomegaly. Normochromic normocytic anemia as well as severe thrombocytopenia—in the face of a hypercellular marrow with increased megakaryocytes and erythroid hyperplasia—were consistent with splenic sequestration. The distinction between SBLPN, splenic diffuse red pulp lymphoma, and SMZL in this case could not be established with confidence as this would require splenectomy with histologic analysis of the spleen—a procedure which was deemed to carry more risks than benefits. Nevertheless, the clinical, immunophenotypic, and hematopathological characteristics were most suggestive of SBLPN.

The clinical course, prognosis, and treatment of SBLPN and splenic lymphomas are distinct from HCL and other lymphoproliferative disorders [4–6]. SBLPN principally affects elderly patients with a median age of 71 years, which is higher than the median age of 62 years for patients with HCL [4]. In our case, the patient was in his 80s, which was in line with the expected epidemiology of SBLPN. Moreover, male predominance is a well‐reported feature of both HCL and splenic lymphomas, and our patient was also male [5]. With regards to the clinical course, SBLPN tends to have a more aggressive clinical course than HCL with a median survival of 9 years [4, 9]. Additionally, the response rate of SBLPN is generally inferior to that for patients with classic HCL [5, 9]. Given the rarity of this condition, guidelines for optimal therapy are less well defined [5]. Based on the available literature, rituximab with or without cladribine is often used for treatment [4, 5]. There is a potential role for Bruton kinase inhibitors, BCl‐2 inhibitors, and pERK inhibitors in the treatment of SBLPN although this remains to be validated through further studies [9]. In our patient, Type I hypersensitivity to rituximab precluded the use of this agent and he was treated with 1 cycle of cladribine. Subsequently, he was switched to obinutuzumab—a humanized anti‐CD20 monoclonal antibody—which has a mechanism of action similar to rituximab with a much lower risk of allergic reactions. Patient tolerated this therapy well and had resolution of fever as well as improvement in cell counts.

In summary, this case highlights SBLPN (formerly known as HCL‐variant) as a rare but important hematologic cause of FUO, particularly in older adults presenting with constitutional symptoms, splenomegaly, and unexplained cytopenias. It also underscores the value of integrating peripheral smear review, flow cytometry, bone marrow examination, and molecular testing when splenic tissue is not feasible, allowing the timely diagnosis of an, otherwise, diagnostically challenging splenic B‐cell neoplasm. The practicing internist needs to be aware of this initial presentation of SBLPN as FUO and have a low suspicion to proceed with a bone marrow aspiration and biopsy to clench the diagnosis in the right clinical context.

## Author Contributions

Abdul Rehman: conceptualization, data curation, writing–original draft, validation, and supervision. John E. McKnight: conceptualization, writing–original draft, validation, and supervision. Hafiz Javed: conceptualization, writing–review and editing, visualization, and validation. Arfa Ahmad: conceptualization, writing–review and editing, and validation. Patrick O’Reilly: conceptualization, writing–review and editing, resources, and supervision.

## Funding

No funding was received for this manuscript.

## Consent

Written informed consent was obtained from the patient by Dr. Abdul Rehman for publication of this case report.

## Conflicts of Interest

The authors declare no conflicts of interest.

## Data Availability

The data that support the findings of this study are available on request from the corresponding author. The data are not publicly available due to privacy or ethical restrictions.
